# Olfactory Bulbectomy Model of Depression Lowers Responding for Food in Male and Female Rats: The Modulating Role of Caloric Restriction and Response Requirement

**DOI:** 10.3390/biomedicines11092481

**Published:** 2023-09-07

**Authors:** Liana Fattore, Petra Amchova, Paola Fadda, Jana Ruda-Kucerova

**Affiliations:** 1 CNR Institute of Neuroscience-Cagliari, National Research Council, 09042 Monserrato, CA, Italy; liana.fattore@in.cnr.it (L.F.); pfadda@unica.it (P.F.); 2 Department of Pharmacology, Faculty of Medicine, Masaryk University, 62500 Brno, Czech Republic; pamchova@med.muni.cz; 3 Department of Biomedical Sciences, Section of Neuroscience and Clinical Pharmacology, University of Cagliari, 09042 Monserrato, CA, Italy

**Keywords:** olfactory bulbectomy, depression, reward, self-administration, sex, strain

## Abstract

Depression is a psychiatric disorder characterized by a marked decrease in reward sensitivity. By using the olfactory bulbectomy (OBX) model of depression, it was shown that OBX rats display enhanced drug-taking and seeking behaviors in a self-administration paradigm than sham-operated (SHAM) controls, and sex is an important regulating factor. To reveal potential strain effects, we compared the operant behavior of male and female Sprague–Dawley and Wistar OBX and SHAM rats trained to self-administer palatable food pellets. Results showed that Sprague–Dawley OBX rats of both sexes exhibited lower operant responding rates and food intake than SHAM controls. Food restriction increased responding in both OBX and SHAM groups. Female rats responded more than males, but the OBX lesion abolished this effect. In Wistar rats, bulbectomy lowered food self-administration only during the last training days. Food self-administration was not significantly affected in Wistar rats by sex. In summary, this study showed that bulbectomy significantly reduces operant responding and food intake in male and female Sprague–Dawley rats while inducing a mild reducing effect only in the Wistar strain. Strain-dependent effects were also observed in the modulating role of sex and food restriction on operant responding and palatable food intake.

## 1. Introduction

Decreased reward responsiveness, weaker encoding of reward value, and greater difficulty in engaging in goal-oriented behaviors increase the risk of developing anhedonic symptoms that may result in loss of motivation. Blunted sensitivity to rewards and lack of motivation are core symptoms of major depressive disorders, potentially life-threatening mental disorders characterized by severe and long-lasting symptoms that make them highly disabling illnesses [1]. Over the years, various animal models have been developed to investigate the pathophysiology of depression and to test novel pharmacological strategies, including genetic and stress models and models based on disruption of the circadian rhythm or pharmacological manipulations [2,3,4].

Olfactory bulbectomy (OBX) is an animal model for major depression that results in neurochemical, behavioral, and neuroendocrine alterations that are reversed by chronic, but not acute, treatment with antidepressants [5,6,7]. Like other animal models of depression, the OBX model shows several phenotypes of depression-related behavior, altered neurotransmitter activity, and neurotransmitter-related gene expression. Yet, it appeared to be characterized by manic-depressive symptoms more than other classical animal models of depression, such as the unpredictable chronic mild stress or the glucocorticoid/corticosterone model [8]. OBX rats have been widely used not only for investigating the molecular, neuroanatomical, and neurobiological bases of depression [9,10] but also for studying its psychiatric comorbidity and isolating the contributing factors [11,12].

Depression and addiction, for example, are frequently comorbid with both directions of causality demonstrated, i.e., depression leading to subsequent drug taking and substance use triggering mood disorders [13,14]. The likelihood of drug addiction and depression occurring together in the same individual is approximately five times greater than expected by the prevalence of each disorder alone [15]. Studies combining the OBX model of depression with the intravenous self-administration procedure showed a higher intake of amphetamine in Sprague–Dawley (SD) OBX rats [16], of methamphetamine and ketamine in OBX Wistar (WI) rats [17,18] and of synthetic cannabinoids in OBX Lister-Hooded (LH) rats [19] with respect to corresponding sham-operated (SHAM) controls. On the other hand, cocaine self-administration in WI rats was not affected by olfactory bulbectomy [20]. In contrast, OBX rats of WI and Long–Evans (LE) strains showed increased oral nicotine intake when tested in the two-bottle choice drinking paradigm [21]. Furthermore, bulbectomy in adult WI rats exposed to alcohol during adolescence significantly altered drinking patterns concerning SHAM control rats [22]. In models of drug-seeking reinstatement after drug abstinence, OBX WI rats showed increased methamphetamine and nicotine [23] but not ketamine [17] seeking behavior and higher drug-induced reinstatement of cocaine seeking [20].

To confirm decreased reward sensitivity in the OBX model, previous studies have demonstrated suppressed sucrose preference after OBX lesion in SD rats [24,25,26,27,28,29,30], LH rats [19], WI rats [31,32,33,34], and LE rats [35]. Yet, negative results were also reported, showing no effect of OBX lesion on sucrose intake in SD [36] and WI rats [37]. Curiously, very few studies on OBX animals were conducted using more than one strain. When this was the case, it turned out that bulbectomy produced quite different neurophysiologic and behavioral alterations in the two strains used [21,38]. The hypothesis that the strain of rats used can represent a potential confounding factor and that outcomes can be different depending on the specific strain used is strengthened by the evidence that rat strain differences exist not only in other animal models of depression [39,40] but also in reward sensitivity [41,42], self-administration of drug [43] and palatable food pellets [44] and in other hedonic behaviors such as social play [45] and the investigatory behavior of a novel conspecific of the opposite sex [46].

Therefore, the present study’s first aim was to verify whether the OBX-induced anhedonic trait could differ depending on the genetic background and whether sensitivity to natural rewards varies between rat strains in the OBX model of depression. To this purpose, we assessed the impact of strain on operant responding for palatable food by using two rat strains commonly used in preclinical research, i.e., SD and WI, which were not used in our previous study [47]. In addition to strain, sex is another factor that has been poorly studied in the OBX model [48], although it is known to affect both depressive-like [49] and reward-related behaviors in rats [50,51,52,53], including OBX rats [47]. In light of this evidence, this study included males and females, and strain x sex interaction was analyzed and discussed. We finally extended our investigation to analyze the feeding conditions (ad libitum vs. restricted diet) on food self-administration in OBX rats.

## 2. Material and Methods

The study was performed in two different laboratories using two different types of operant responses, namely lever-pressing and nose-poking, where animals were required to press a lever or to put their nose into a specific hole to obtain a contingent food pellet. Although the studies were performed at different sites, the laboratories have collaborated for over a decade and established the OBX procedure together. Experiment 1 with Sprague–Dawley (SD) rats and lever-pressing as *operandum* was performed in the University of Cagliari (Italy, IT), while Experiment 2 with Wistar (WI) rats and nose-poking as *operandum* was performed in the Masaryk University, Czech Republic (CZ). Notably, both surgeries (i.e., OBX and insertion of the intravenous catheter) and self-administration training were performed by the same experimenters in the two laboratories.

### 2.1. Animals

Twenty-four male and twenty-four female SD rats (weight range 225–250 g upon arrival) were purchased from Harlan-Nossan (Italy) and housed 4 per cage at the Animal Facility of the Department of Biomedical Sciences, University of Cagliari, IT. Fifty male and thirty-two female WI rats (weight range 225–250 g upon arrival) were purchased from the Masaryk University breeding facility and housed 4 per cage at the Central Animal Facility of the Faculty of Medicine, Masaryk University, CZ. Males and females were housed in different rooms with free access to water, while food was available either *ad libitum* or restricted to 18 g/day for the food-restricted cohorts to reach approximately 80% of the free-feeding body weight. Environmental enrichment was provided throughout the study to all animals. Environmental conditions during the whole study were constant: relative humidity 50–60%, room temperature 23 ± 1 °C, inverted 12 h light–dark cycle (7 a.m. to 7 p.m. darkness). All procedures were performed following EU Directive No. 2010/63/EU and approved by the Animal Care Committee of the Faculty of Medicine, Masaryk University, Czech Republic and Czech Governmental Animal Care Committee, in compliance with Czech Animal Protection Act No. 246/1992.

### 2.2. Olfactory Bulbectomy Surgery

At the beginning of the experiments, rats were randomly divided into two equal groups: bulbectomized (OBX) and sham-operated (SHAM) rats. The bilateral ablation of the olfactory bulbs was performed as previously described [17,19,54]. Animals were anesthetized with isoflurane 2% (SD rats, IT) or ketamine 50 mg/kg and xylazine 8 mg/kg given intraperitoneally (WI rats, CZ); the top of the skull was shaved and swabbed with an antiseptic solution. Then, a midline frontal incision was made on the skull, and the skin was retracted bilaterally. Two burr holes, 2 mm in diameter, were drilled in the frontal bone 7 and 7.5 mm anterior from the bregma, 1.5 and 2 mm lateral to bregma suture for rats weighing 230 ± 10 g and 260 ± 10 g, respectively. Both olfactory bulbs were removed by aspiration, paying particular attention to not damaging the frontal cortex. Prevention of blood loss from the ablation cavity was achieved by filling the dead space with a hemostatic sponge. The skin above the lesion was closed with a suture. Finally, bacitracin plus neomycin powder was applied to prevent bacterial infection. Sham-operated rats underwent identical anesthetic and drilling procedures, but their bulbs were left intact. A period of at least 20 days was allowed to recover from the surgical procedure and develop the characteristic phenotype. During this period, animals were handled regularly to eliminate aggression, which could otherwise arise [5,55]. At the end of the study, rats were euthanized by an aesthetic overdose, and the brains were dissected to confirm the successful removal of the olfactory bulbs. Animals with incomplete removal of the olfactory bulbs were eliminated from the analysis: specifically, 6 SD males, 9 SD females, 11 WI males and 7 WI females were not included.

### 2.3. Food Self-Administration Apparatus and Protocol

**Experiment 1 (SD):** Food self-administration was conducted in 12 operant chambers (29.5 × 32.5 × 23.5 cm, Med Associates, VT, USA) using lever pressing as an *operandum*. Each chamber was encased in a sound and light-attenuating cube and provided a ventilation fan and a front panel equipped with two retractable levers (each 4 cm wide) positioned 12 cm apart 8 cm from the grid, and 1.5 cm into the box. A white house light illuminated the cage during the whole session.

**Experiment 2 (WI):** Food self-administration was conducted in 10 operant boxes (30 × 25 × 30 cm, Coulbourn Instruments, Allentown, NJ, USA) using a nose-poke *operandi* encased in a sound and light attenuating cube. Each cage was provided with two nose-poke holes allocated on one side and programmed by the software Graphic State Notation 3.03 (Coulbourn Instruments, Allentown, NJ, USA). A white house light illuminated the cage during the whole session.

In both experiments, the training was conducted under a fixed ratio 1 (FR1) schedule of reinforcement, i.e., the animal had to press the active lever or break a photobeam by putting its nose into a hole (i.e., by acting an active nose-poke) once to obtain a single palatable pellet (BioServ, sweet dustless rodent pellets, F0021-Purified Casein Based Formula—45 mg). After each active lever-press, both levers were retracted for a 15 s timeout period. Stimulation of the inactive operandum was recorded but did not lead to any programmed consequence. The session lasted 30 min, and the house light was on throughout. The length of the training was 10 consecutive days. All animals consumed the vast majority of the gained pellets. All self-administration sessions were conducted at the same time daily (10 a.m.–12.30 p.m.) during the dark period of the inverted light–dark cycle.

### 2.4. Statistical Analysis

Primary data were summarized using arithmetic mean and standard error of the mean estimate (SEM). The analyses were calculated using Statistica 13.2 (StatSoft, Tulsa, OK, USA). A value of *p* < 0.05 was recognized as a boundary of statistical significance in all applied tests. Food self-administration data were analyzed by ANOVA for repeated measures (RM) with factors: OBX model, feeding status, repeated variable: day, followed by Bonferroni’s post-test for analysis of significant interactions of factors. Sex differences in the food self-administration were also assessed (RM) ANOVA (factors: OBX model, sex, repeated variable: day) followed by Bonferroni’s post hoc test for analysis of significant interactions of factors. In this analysis, the factor of the feeding status was not included, as we observed the same effect of food restriction in male and female rats of both strains.

## 3. Results

### 3.1. Experiment 1 (SD)

In Experiment 1, we evaluated the self-administration of palatable food pellets over 10 consecutive days in male and female SD rats. Data are presented as mean active and inactive lever presses and as a cumulative number of pellets gained over time, i.e., day 1: number of pellets delivered on day 1; day 2: sum of the pellets obtained on day 1 + those obtained on day 2; day 3: sum of the pellets obtained on day 1 + day 2 + day 3, etc).

#### 3.1.1. Male SD Rats

In active lever pressing, RM ANOVA revealed a highly significant effect of the OBX model (F_1,20_ = 29.48, *p* < 0.001), feeding status (F_1,20_ = 5.69, *p =* 0.027), day (F_9,180_ = 45.52, *p* < 0.001) and day*OBX model interaction (F_9,180_ = 10.54, *p* < 0.001). According to the Bonferroni’s post hoc test for day*OBX model interaction, active responding in the OBX rats resulted significantly decreased from day 6 onwards (days 6: p = 0.004, days 7–10: *p* < 0.001), while inactive lever pressing did not differ among the groups. RM ANOVA detected a significant effect of the repeated factor—day only (F_9,180_ = 5.81, *p* < 0.001). Significant effects of a day in both active and inactive operant responding reflect the learning process during acquisition, which is irrelevant to this study.

**Figure 1:**
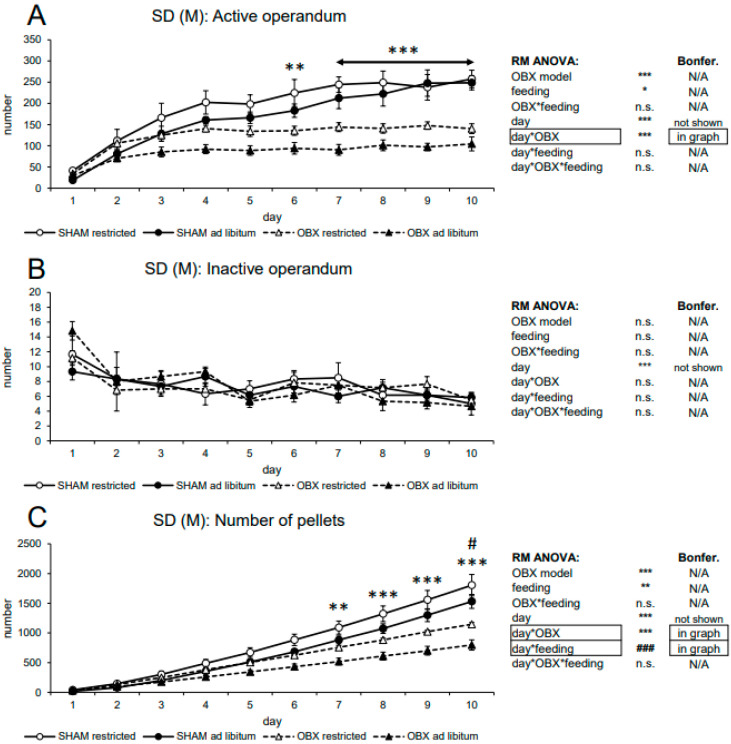
Self-administration of sweet pellets in male SD rats. The graphs present the mean ± SEM of daily numbers of active lever presses (**A**), inactive lever presses (**B**), and cumulative numbers of self-administered pellets (**C**) in all experimental groups. The tables summarize the results of RM ANOVA and Bonferroni’s post-test for each variable (* *p* < 0.05, ** *p* < 0.01, *** *p* < 0.001 and ^#^ *p* < 0.05, ^###^ *p* < 0.001 for day*feeding interaction). N/A: not applicable; n.s.: not significant.

The cumulative number of delivered pellets showed similar trends as active responding. RM ANOVA showed a highly significant effect of the OBX model (F_1,20_ = 19.23, *p* < 0.001), feeding status (F_1,20_ = 8.24, *p* = 0.009), day (F_9,180_ = 368.10, *p* < 0.001), day*OBX model interaction (F_9,180_ = 28.78, *p* < 0.001) and day*feeding status interaction (F_9,180_ = 4.90, *p* < 0.001). Bonferroni’s post hoc test for day*OBX model interaction revealed significantly suppressed pellet intake in OBX rats from day 7 onwards (day 7: *p* = 0.005, days 8–10: *p* < 0.001). Bonferroni’s post hoc test for day*feeding status interaction revealed higher pellet intake in the food-restricted animals on day 10 (*p =* 0.024). The significant effect of day in pellet delivery reflects the learning process during acquisition, which is irrelevant to this study. All variables are shown in Figure 1 alongside an overview of statistical results.

#### 3.1.2. Female SD Rats

In active lever pressing, RM ANOVA revealed a highly significant effect of the OBX model (F_1,20_ = 155.44, *p* < 0.001), feeding status (F_1,20_ = 6.54, *p =* 0.019), day (F_9,180_ = 51.45, *p* < 0.001) and day*OBX model interaction (F_9,180_ = 13.31, *p* < 0.001). Bonferroni’s *post hoc* test for day*OBX model interaction indicated significantly lower responding in the OBX rats from day 3 onwards (*p* < 0.001). Interestingly, in inactive lever pressing, RM ANOVA indicated not only a significant effect of the day (F_9,180_ = 7.16, *p* < 0.001) but also significant day*feeding status interaction (F_9,180_ = 2.22, *p* = 0.023) and day*OBX model*feeding status interaction (F_9,180_ = 1.96, *p* = 0.047). However, Bonferroni’s *post hoc* tests for the interactions did not reveal any relevant differences.

**Figure 2:**
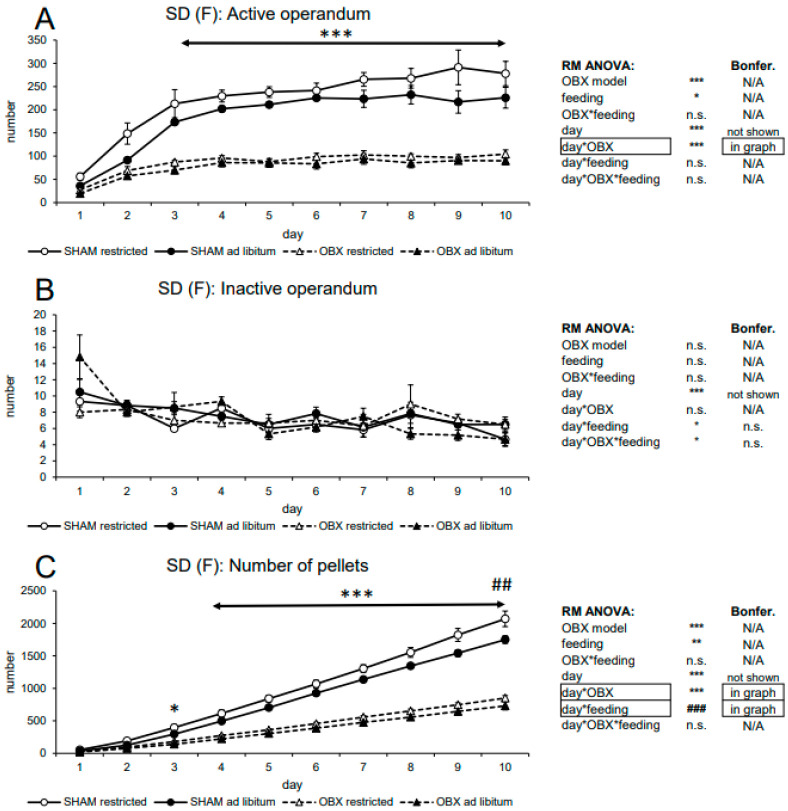
Self-administration of sweet pellets in female SD rats. The graphs present the mean ± SEM of daily numbers of active lever presses (**A**), inactive lever presses (**B**), and cumulative numbers of self-administered pellets (**C**) in all experimental groups. The tables summarize the results of RM ANOVA and Bonferroni’s post-test for each variable (* *p* < 0.05, ** *p* < 0.01, *** *p* < 0.001 and ^##^ *p* < 0.01, ^###^ *p* < 0.001 for day*feeding interaction). N/A: not applicable; n.s.: not significant.

In the analysis of the cumulative number of delivered pellets, RM ANOVA showed a highly significant effect of the OBX model (F_1,20_ = 198.02, *p* < 0.001), feeding status (F_1,20_ = 8.67, *p =* 0.008), day (F_9,180_ = 951.06, *p* < 0.001), day*OBX model interaction (F_9,180_ = 169.66, *p* < 0.001) and day*feeding status interaction (F_9,180_ = 4.73, *p* < 0.001). Food pellet intake was significantly decreased in the OBX rats from day 3 onwards (day 3: *p =* 0.044, days 4–10: *p* < 0.001, Bonferroni’s *post hoc* test for day*OBX model interaction). Conversely, food pellet intake was higher in the food-restricted animals on days 9 (*p =* 0.030) and 10 (*p =* 0.004, Bonferroni’s *post hoc* test for day*feeding status interaction). All variables are shown in Figure 2 together with an overview of statistical results.

#### 3.1.3. Evaluation of Sex Differences in SD Rats

**Figure 3:**
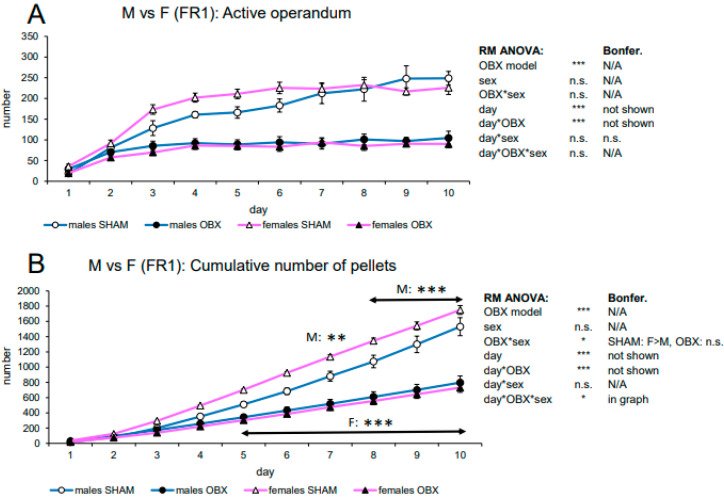
Sex differences in the self-administration of sweet pellets in SD rats. The graphs present the mean ± SEM of daily numbers of active lever presses (**A**) and cumulative numbers of self-administered pellets (**B**) in ad libitum-fed experimental groups of both sexes. The tables summarize the results of RM ANOVA and Bonferroni’s post-test for each variable (* *p* < 0.05, ** *p* < 0.01, *** *p* < 0.001). N/A: not applicable; n.s.: not significant.

Analysis of sex differences included ad libitum-fed groups only, i.e., analyzed groups were males SHAM, males OBX, females SHAM, and females OBX, while variables included active lever presses and cumulative pellet intake (Figure 3).

In active lever pressing, RM ANOVA revealed a significant effect of the OBX model (F_1,20_ = 84.94, *p* < 0.001), day (F_9,180_ = 59.26, *p* < 0.001), and day*OBX model interaction (F_9,180_ = 17.00, *p* < 0.001) but no effect of sex. Bonferroni’s post hoc test for day*OBX model interaction indicated significantly lower responding in the OBX rats from day 3 onwards (day 3: *p* = 0.001, day 4–10: *p* < 0.001), which is consistent with findings in both sexes.

In the cumulative number of delivered pellets, RM ANOVA showed a highly significant effect of the OBX model (F_1,20_ = 91.60, *p* < 0.001) and OBX model*sex interaction (F_1,20_ = 7.46, *p* = 0.013). Bonferroni’s post hoc test for OBX model*sex interaction indicated a significantly higher intake of food pellet in female SHAM rats as compared to the corresponding male group (*p* = 0.032) but not in female OBX rats as compared to male OBX rats (n.s.). As expected, the difference between OBX and SHAM groups within each sex was also present: SHAM rats consumed more pellets than the OBX group (*p* < 0.001 in both sexes). Furthermore, as expected, RM ANOVA indicated a significant effect of the day (F_9,180_ = 554.48, *p* < 0.001), day*OBX model interaction (F_9,180_ = 80.35, *p* < 0.001) but also a day*OBX model*sex interaction (F_9,180_ = 2.36, *p* = 0.015). Bonferroni’s post hoc test for day*OBX model interaction revealed a decreased number of pellets in the OBX animals from day 5 onwards (*p* < 0.001). Bonferroni’s post hoc test for the day*OBX model*sex interaction revealed that the effect of OBX in male rats is present from day 7 (*p* = 0.004) until day 10 (day 8–10, *p* < 0.001), while in female rats, this effect is apparent already on day 5 (day 5–10, *p* < 0.001).

### 3.2. Experiment 2 (WI)

Experiment 2 assessed an operant self-administration of palatable pellets over 10 days under the FR1 schedule of reinforcement in male and female WI rats. Data are presented as daily mean numbers of active nose-poking, inactive nose-poking, and cumulative number of delivered pellets (i.e., as in Experiment 1).

#### 3.2.1. Male WI Rats

**Figure 4:**
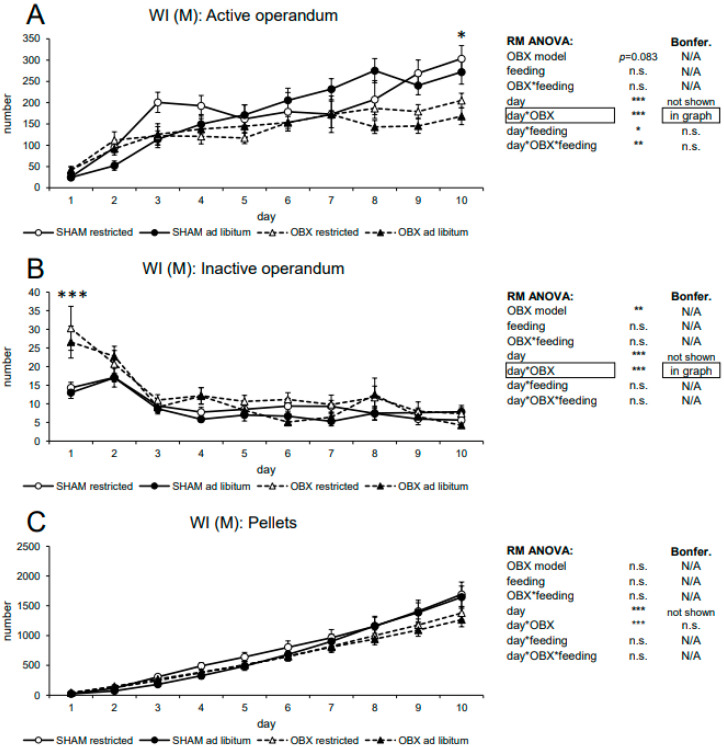
Self-administration of sweet pellets in male WI rats. The graphs present the mean ± SEM of daily numbers of active lever presses (**A**), inactive lever presses (**B**), and cumulative numbers of self-administered pellets (**C**) in all experimental groups. The tables summarize the results of RM ANOVA and Bonferroni’s post-test for each variable (* *p* < 0.05, ** *p* < 0.01, *** *p* < 0.001). N/A: not applicable; n.s.: not significant.

In active nose-poking RM ANOVA revealed only a trend to an effect of the OBX model (F_1,45_ = 3.14, *p =* 0.083) but a significant effect of the day (F_9,405_ = 51.07, *p* < 0.001), day*OBX model interaction (F_9,405_ = 5.97, *p* < 0.001), day*feeding status interaction (F_9,405_ = 2.37, *p =* 0.013) and day*OBX model*feeding status interaction (F_9,405_ = 3.11, *p* = 0.001). Bonferroni’s post hoc test for day*OBX model interaction indicated significantly decreased responding in the OBX rats on day 10 (*p* = 0.038). Bonferroni’s post hoc test for other interactions did not detect any significant results. In inactive nose-poking, RM ANOVA revealed a significant effect of the OBX model (F_1,45_ = 9.79, *p* = 0.003), day (F_9,405_ = 25.10, *p* < 0.001), and day*OBX model interaction (F_9,405_ = 4.97, *p* < 0.001). Bonferroni’s post hoc test for day*OBX model interaction indicated significantly increased responding in the OBX rats on day 1 (*p* < 0.001). This corresponds to our earlier findings showing higher inactive nose-poke responding in the OBX model, which was likely due to novelty-induced hyperactivity [17,47]. In the cumulative number of delivered pellets, RM ANOVA did not detect a significant effect of the OBX model but only day (F_9,405_ = 248.86, *p* < 0.001) and day*OBX model interaction (F_9,405_ = 4.18, *p* < 0.001). However, Bonferroni’s post hoc test for day*OBX model interaction did not reveal significant results. All variables are shown in Figure 4 together with an overview of statistical results.

#### 3.2.2. Female WI Rats

**Figure 5:**
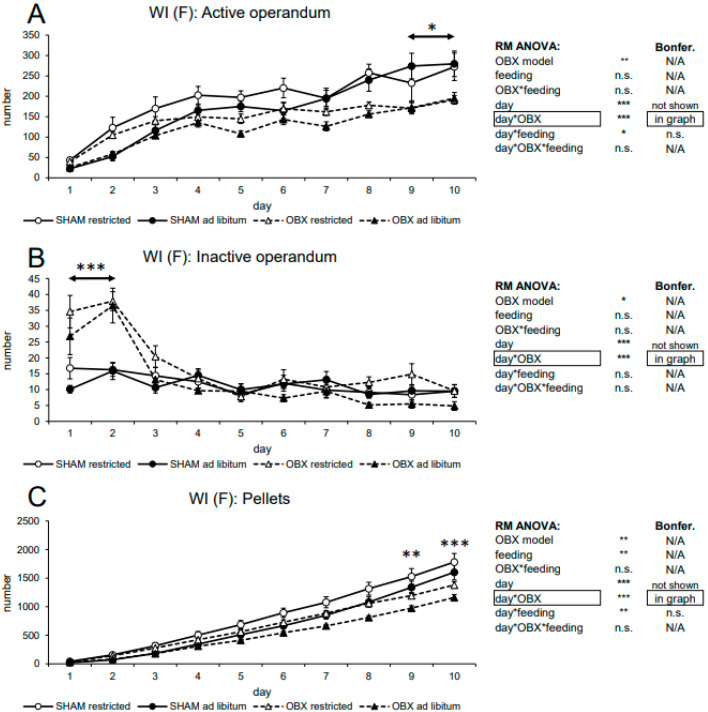
Self-administration of sweet pellets in FEMALE WI rats. The graphs present the mean ± SEM of daily numbers of active lever presses (**A**), inactive lever presses (**B**), and cumulative numbers of self-administered pellets (**C**) in all experimental groups. The tables summarize the results of RM ANOVA and Bonferroni’s post-test for each variable (* *p* < 0.05, ** *p* < 0.01, *** *p* < 0.001). N/A: not applicable; n.s.: not significant.

In active nose-poking, RM ANOVA revealed a significant effect of the OBX model (F_1,26_ = 10.76, *p =* 0.003), day (F_9,234_ = 54.21, *p* < 0.001), day*OBX model interaction (F_9,234_ = 3.32, *p* < 0.001) and day*feeding status interaction (F_9,234_ = 2.08, *p* = 0.032). Bonferroni’s *post hoc* test for day*OBX model interaction indicated significantly lower responding in the OBX rats on days 9 and 10 (*p* = 0.028). For the day*feeding status interaction, Bonferroni’s *post hoc* test did not show any relevant differences. In inactive nose-poking RM ANOVA revealed an effect of the OBX model (F_1,26_ = 7.35, *p* = 0.012), day (F_9,234_ = 22.47, *p* < 0.001), and day*OBX model interaction (F_9,234_ = 11.07, *p* < 0.001). Bonferroni’s *post hoc* test for day*OBX model interaction indicated significantly increased responding in the OBX rats on days 1–2 (*p* < 0.001) analogously as in male WI rats and our previous studies [17,47]. In the analysis of the cumulative number of delivered pellets, RM ANOVA showed a significant effect of OBX model (F_1,28_ = 7.96, *p* = 0.009), feeding status (F_1,28_ = 8.12, *p* = 0.008), day (F_9,252_ = 536.01, *p* < 0.001), day*OBX model interaction (F_9,252_ = 12.07, *p* < 0.001) and day*feeding status interaction (F_9,252_ = 2.84, *p* = 0.003). Bonferroni’s post-hoc test for day*OBX model interaction indicated significantly decreased pellet intake in the OBX rats on days 9 (*p* = 0.002) and 10 (*p* < 0.001). Bonferroni’s *post hoc* test for day*feeding status interaction did not show significant differences. All variables are shown in Figure 5 together with an overview of statistical results.

#### 3.2.3. Evaluation of Sex Differences in WI Rats

**Figure 6:**
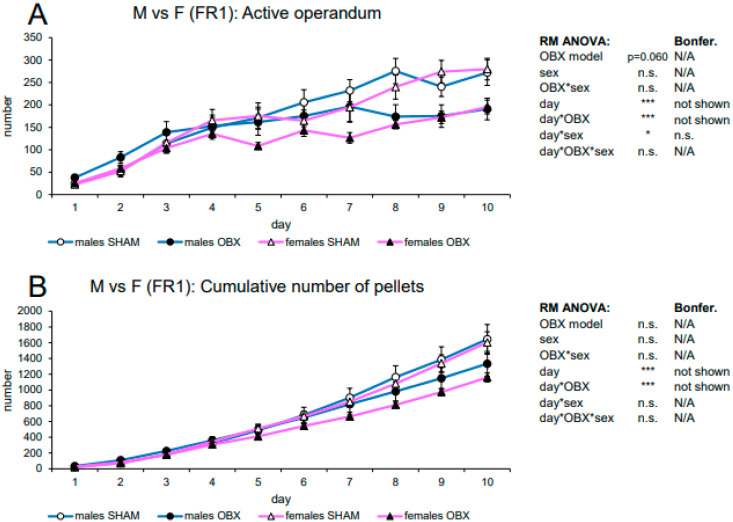
Sex differences in the self-administration of sweet pellets in WI rats. The graphs present the mean ± SEM of daily numbers of active nose-pokes (**A**) and cumulative numbers of self-administered pellets (**B**) in ad libitum-fed experimental groups of both sexes. The tables summarize RM ANOVA and Bonferroni’s post-test results for each variable (* *p* < 0.05, *** *p* < 0.001). N/A: not applicable; n.s.: not significant.

The analysis of sex differences included *ad libitum* fed groups only and the number of active nose-pokes and cumulative pellets as variables (Figure 6). In active nose-poking activity, RM ANOVA revealed only a trend to a significant effect of the OBX model (F_1,35_ = 3.79, *p* = 0.060) but indicated a significant effect of the day (F_9,315_ = 68.68, *p* < 0.001), day*OBX model interaction (F_9,315_ = 9.16, *p* < 0.001) and day*sex interaction (F_9,315_ = 1.93, *p* = 0.047). Bonferroni’s *post hoc* test for day*OBX model interaction indicated significantly lower responding in the OBX rats compared to SHAM on days 8 (*p* = 0.001), 9 (*p* = 0.031), and 10 (*p* = 0.032). Bonferroni’s *post hoc* test for day*sex interaction showed no relevant significant results. In the cumulative number of delivered pellets, RM ANOVA showed a highly significant effect of the day (F_9,315_ = 252.66, *p* < 0.001) and day*OBX model interaction (F_9,315_ = 7.12, *p* < 0.001). Bonferroni’s *post hoc* test for day*OBX model interaction confirmed a decreased number of pellets in the OBX animals on day 10 (*p =* 0.014).

## 4. Discussion

Overall, the present study confirmed that OBX reduced food self-administration in male and female rats and demonstrated that sex does not seem to play a major role in the OBX-induced changes in food self-administration behavior. Notably, we observed a relatively consistent influence of OBX on the food self-administration behavior in two different rat strains (i.e., SD and WI).

In general, data obtained in SD rats align with those we previously reported in LH rats [47], as OBX lesion decreased active responding and food intake in both sexes, and the restricted diet condition moderately increased food pellet intake in both males and females. Actually, food restriction is known to facilitate the acquisition and maintenance of self-administration of most abused drugs, so we expected that palatable food acts as a more effective reinforcer when animals are food deprived [56,57]. Contrary to the LH strain, however, we found that in the SD strain, the effect of sex is evident in SHAM animals only, as OBX SD male and female rats did not significantly differ in their active responding or the cumulative number of pellets. Notably, the finding that female SD rats self-administered by lever pressing more pellets than SD males is in line with another previous study [58] where a nose-poking operandum was used, which ensures that such a sex-dependent effect does not depend upon the specific response requirement.

In the WI strain, the effect of OBX was relatively weak, more robust in females than in males, but still weaker than in SD rats, and with no significant sex-dependent effects. These observations would suggest that WI rats could not represent the best strain to investigate the co-occurrence of depression and motivated or reward-related behavior when using animals of both sexes, as this rat strain might not reveal potential underlying sex-dependent effects. Notably, although WI rats displayed sex-dependent differences in the self-administration of palatable food [59,60], no study at present has used this strain in the OBX model of food self-administration, and none of the previous studies investigating operant responding for drugs in OBX WI rats used animals of both sexes [18,20,61,62]. The present study is thus the first to test WI OBX rats in the food self-administration paradigm and to evaluate potential sex differences in responding for food. With respect to the SD strain, WI rats have been reported to display a weaker response to another natural reward, i.e., social play [43], suggesting a different reward sensitivity in the two strains, which could explain the differences we observed in their response to palatable food. It is noteworthy that in line with our previous observation in this strain [17], both female and male WI OBX rats displayed higher inactive responding than WI SHAM rats during the first day of training, suggesting the occurrence of hyperactivity as a consequence of bulbectomy. This effect was not detectable in the SD cohort likely due to the use of levers instead of nose pokes. Indeed, while the nose-poking mechanism is easier to stimulate repeatedly as photobeams are constantly present and available to rats, levers retracted after each active press, making it more difficult to appreciate potential hypermotility.

The reducing effect of OBX on active responding was less evident in WI than in SD rats, which is similar to the effect of the ad libitum food condition that significantly reduced food intake in both SHAM and OBX rats in the SD but not the WI strain.

Motivation for food is regulated by both homeostatic and hedonic mechanisms, so we included the feeding status factor in our study to assess whether satiety may interfere with the reward value of the hedonic food and affect the operant responding of rats. Our findings demonstrated that OBX affects the motivational effects of hedonic food rewards independently of the homeostatic needs, i.e., in both the “fed ad libitum” and the “restricted diet” groups. As expected, food restriction consistently increased food self-administration in both SHAM and OBX rats [63], the difference between ad libitum vs. restricted food conditions becoming more evident as the training days went by. Yet, we did not detect OBX*feeding status interaction on either strain or sex, which suggests that this factor can be excluded from future studies, as it affects all experimental groups consistently.

Importantly, although not always reaching a statistically significant level, a more evident OBX-induced reduction in food self-administration was observed in female than in male SD rats, suggesting that besides the strain, also sex should be considered an important variable in the OBX model [47]. In addition to direct strain-dependent effects, protocol-dependent differences may also play a role in the reported findings, with WI rats (in which OBX showed a minor effect) accessing food pellets through nose poking, which requires less motor and motivational output than lever-pressing. WI rats were also able to nose poke more than once at a time unintentionally or to continue nose poking intentionally in the active hole until the presentation of the food pellet (i.e., even during the delivery time), which are events that are both not possible for SD rats that operated through levers that retracted immediately after each active lever press. These small protocol differences could explain (at least in part) the relatively higher interindividual variability of active responding in WI vs. SD rats, with the latter showing higher responding for food by operating through a modality that typically requires more motor and motivational outputs.

Interestingly, while other studies that used the OBX model for investigating depression and drug abuse comorbidity reported enhanced responding for amphetamine [16], methamphetamine [18], cannabinoid [19], and ketamine [17], the present study confirms that the opposite occurs when OBX rats are given free access to a natural rewarding stimulus like palatable food [47]. This evidence suggests that while the neurochemical abnormalities associated with depression may enhance the addictive properties of some drugs of abuse, although not all [61,62,64], they may also render the subjects less responsive to other types of reinforcers. Yet, the possibility that OBX rats self-administer some drugs more than SHAM animals to alleviate the depression-like symptoms cannot be ruled out [21].

In conclusion, this study demonstrates for the first time that while there are some strain-dependent differences in operant responding for food, the OBX lesion exerts changes, which are relatively consistent among the rat strains and sexes. Mainly, our data confirm that OBX reduces the self-administration of palatable food in rats and that its effect may vary significantly between males and females. Our findings suggest that innate differences in susceptibility to develop depression-like symptoms after OBX may be present between rat strains. Lever pressing could represent the most useful response-like operandum when testing male and female OBX rats in self-administration paradigms.

Yet, some limitations of this study should be considered. Despite our efforts to minimize differences between the two laboratories where the two strains of rats have been tested, including having the same person in charge of OBX surgeries and training, a “lab effect” cannot be excluded. Notably, our purely behavioral approach does not offer any mechanistic insight into the strain- and sex-induced effects. However, it does not affect the main take-home message of this study, i.e., to consider the rat strain, the response-like requirement, and the animals’ sex when using the OBX model for reward-related studies.

## Data Availability

The data presented in this study are available on request from the corresponding author.

## References

[1] Wang S., Leri F., Rizvi S.J. (2021). Anhedonia as a Central Factor in Depression: Neural Mechanisms Revealed from Preclinical to Clinical Evidence. Prog. Neuropsychopharmacol. Biol. Psychiatry.

[2] Hao Y., Ge H., Sun M., Gao Y. (2019). Selecting an Appropriate Animal Model of Depression. Int. J. Mol. Sci..

[3] Harro J. (2019). Animal Models of Depression: Pros and Cons. Cell Tissue Res..

[4] Czeh B., Fuchs E., Wiborg O., Simon M. (2015). Animal Models of Major Depression and Their Clinical Implications. Prog. Neuropsychopharmacol. Biol. Psychiatry.

[5] Song C., Leonard B.E. (2005). The Olfactory Bulbectomised Rat as a Model of Depression. Neurosci. Biobehav. Rev..

[6] Harkin A., Kelly J.P., Leonard B.E. (2003). A Review of the Relevance and Validity of Olfactory Bulbectomy as a Model of Depression. Clin. Neurosci. Res..

[7] Abelaira H.M., Réus G.Z., Quevedo J. (2013). Animal Models as Tools to Study the Pathophysiology of Depression. Rev. Bras. Psiquiatr..

[8] Zhu H., Tao Y., Wang T., Zhou J., Yang Y., Cheng L., Zhu H., Zhang W., Huang F., Wu X. (2020). Long-Term Stability and Characteristics of Behavioral, Biochemical, and Molecular Markers of Three Different Rodent Models for Depression. Brain Behav..

[9] Morales-Medina J.C., Iannitti T., Freeman A., Caldwell H.K. (2017). The Olfactory Bulbectomized Rat as a Model of Depression: The Hippocampal Pathway. Behav. Brain Res..

[10] Rajkumar R., Dawe G.S. (2018). OBscure but Not OBsolete: Perturbations of the Frontal Cortex in Common between Rodent Olfactory Bulbectomy Model and Major Depression. J. Chem. Neuroanat..

[11] Wang D., Noda Y., Tsunekawa H., Zhou Y., Miyazaki M., Senzaki K., Nabeshima T. (2007). Behavioural and Neurochemical Features of Olfactory Bulbectomized Rats Resembling Depression with Comorbid Anxiety. Behav. Brain Res..

[12] Filip M., Frankowska M., Jastrzebska J., Wydra K., Przegalinski E. (2013). Preclinical Studies on Comorbidity between Depression and Psychostimulant Addiction. Pharmacol. Rep..

[13] Lieb R., Dom G., Moggi F. (2015). Epidemiological Perspectives on Comorbidity Between Substance Use Disorders and Other Mental Disorders. Co-Occurring Addictive and Psychiatric Disorders: A Practice-Based Handbook from a European Perspective.

[14] Torrens M., Rossi P., Dom G., Moggi F. (2015). Mood Disorders and Addiction. Co-Occurring Addictive and Psychiatric Disorders. A Practice-Based Handbook from a European Perspective.

[15] Ortiz-Gomez L.D., Lopez-Canul B., Arankowsky-Sandoval G. (2014). Factors Associated with Depression and Suicide Attempts in Patients Undergoing Rehabilitation for Substance Abuse. J. Affect. Disord..

[16] Holmes P.V., Masini C.V., Primeaux S.D., Garrett J.L., Zellner A., Stogner K.S., Duncan A.A., Crystal J.D. (2002). Intravenous Self-Administration of Amphetamine Is Increased in a Rat Model of Depression. Synapse.

[17] Babinska Z., Ruda-Kucerova J. (2017). Differential Characteristics of Ketamine Self-Administration in the Olfactory Bulbectomy Model of Depression in Male Rats. Exp. Clin. Psychopharmacol..

[18] Kucerova J., Pistovcakova J., Vrskova D., Dusek L., Sulcova A. (2012). The Effects of Methamphetamine Self-Administration on Behavioural Sensitization in the Olfactory Bulbectomy Rat Model of Depression. Int. J. Neuropsych..

[19] Amchova P., Kucerova J., Giugliano V., Babinska Z., Zanda M., Scherma M., Dusek L., Fadda P., Micale V., Sulcova A. (2014). Enhanced Self-Administration of the CB1 Receptor Agonist WIN55,212-2 in Olfactory Bulbectomized Rats: Evaluation of Possible Serotonergic and Dopaminergic Underlying Mechanisms. Front. Pharmacol..

[20] Frankowska M., Jastrzebska J., Nowak E., Bialko M., Przegalinski E., Filip M. (2014). The Effects of N-Acetylcysteine on Cocaine Reward and Seeking Behaviors in a Rat Model of Depression. Behav. Brain Res..

[21] Vieyra-Reyes P., Mineur Y.S., Picciotto M.R., Tunez I., Vidaltamayo R., Drucker-Colin R. (2008). Antidepressant-like Effects of Nicotine and Transcranial Magnetic Stimulation in the Olfactory Bulbectomy Rat Model of Depression. Brain Res. Bull..

[22] Siska F., Amchova P., Kuruczova D., Tizabi Y., Ruda-Kucerova J. (2021). Effects of Low-Dose Alcohol Exposure in Adolescence on Subsequent Alcohol Drinking in Adulthood in a Rat Model of Depression. World J. Biol. Psychiatry.

[23] Babinska Z., Ruda-Kucerova J., Amchova P., Merhautova J., Dusek L., Sulcova A. (2016). Olfactory Bulbectomy Increases Reinstatement of Methamphetamine Seeking after a Forced Abstinence in Rats. Behav. Brain Res..

[24] Lucas G., Rymar V.V., Du J., Mnie-Filali O., Bisgaard C., Manta S., Lambas-Senas L., Wiborg O., Haddjeri N., Piñeyro G. (2007). Serotonin(4) (5-HT(4)) Receptor Agonists Are Putative Antidepressants with a Rapid Onset of Action. Neuron.

[25] Padilla K.M., Quintanar-Setephano A., López-Vallejo F., Berumen L.C., Miledi R., García-Alcocer G. (2018). Behavioral Changes Induced through Adenosine A2A Receptor Ligands in a Rat Depression Model Induced by Olfactory Bulbectomy. Brain Behav..

[26] Primeaux S.D., Wilson M.A., Wilson S.P., Guth A.N., Lelutiu N.B., Holmes P.V. (2003). Herpes Virus-Mediated Preproenkephalin Gene Transfer in the Ventral Striatum Mimics Behavioral Changes Produced by Olfactory Bulbectomy in Rats. Brain Res..

[27] Romeas T., Morissette M.C., Mnie-Filali O., Pineyro G., Boye S.M. (2009). Simultaneous Anhedonia and Exaggerated Locomotor Activation in an Animal Model of Depression. Psychopharmacology.

[28] Shin M.-S., Park S.-S., Lee J.-M., Kim T.-W., Kim Y.-P. (2017). Treadmill Exercise Improves Depression-like Symptoms by Enhancing Serotonergic Function through Upregulation of 5-HT1A Expression in the Olfactory Bulbectomized Rats. J. Exerc. Rehabil..

[29] Xu J., Xu H., Liu Y., He H., Li G. (2015). Vanillin-Induced Amelioration of Depression-like Behaviors in Rats by Modulating Monoamine Neurotransmitters in the Brain. Psychiatry Res..

[30] Zhang X., Du Q., Liu C., Yang Y., Wang J., Duan S., Duan J. (2016). Rhodioloside Ameliorates Depressive Behavior via Up-Regulation of Monoaminergic System Activity and Anti-Inflammatory Effect in Olfactory Bulbectomized Rats. Int. Immunopharmacol..

[31] Gupta D., Radhakrishnan M., Thangaraj D., Kurhe Y. (2014). Antidepressant and Anti-Anxiety like Effects of 4i (N-(3-Chloro-2-Methylphenyl) Quinoxalin-2-Carboxamide), a Novel 5-HT3 Receptor Antagonist in Acute and Chronic Neurobehavioral Rodent Models. Eur. J. Pharmacol..

[32] Jindal A., Mahesh R., Bhatt S. (2014). Etazolate, a Phosphodiesterase-4 Enzyme Inhibitor Produces Antidepressant-like Effects by Blocking the Behavioral, Biochemical, Neurobiological Deficits and Histological Abnormalities in Hippocampus Region Caused by Olfactory Bulbectomy. Psychopharmacology.

[33] Jindal A., Mahesh R., Bhatt S. (2015). Type 4 Phosphodiesterase Enzyme Inhibitor, Rolipram Rescues Behavioral Deficits in Olfactory Bulbectomy Models of Depression: Involvement of Hypothalamic-Pituitary-Adrenal Axis, CAMP Signaling Aspects and Antioxidant Defense System. Pharmacol. Biochem. Behav..

[34] Rinwa P., Kumar A. (2014). Panax Quinquefolium Involves Nitric Oxide Pathway in Olfactory Bulbectomy Rat Model. Physiol. Behav..

[35] Chambliss H.O., Van Hoomissen J.D., Holmes P.V., Bunnell B.N., Dishman R.K. (2004). Effects of Chronic Activity Wheel Running and Imipramine on Masculine Copulatory Behavior after Olfactory Bulbectomy. Physiol. Behav..

[36] Slattery D.A., Markou A., Cryan J.F. (2007). Evaluation of Reward Processes in an Animal Model of Depression. Psychopharmacology.

[37] Stepanichev M., Markov D., Pasikova N., Gulyaeva N. (2016). Behavior and the Cholinergic Parameters in Olfactory Bulbectomized Female Rodents: Difference between Rats and Mice. Behav. Brain Res..

[38] Otmakhova N.A., Gurevich E.V., Katkov Y.A., Nesterova I.V., Bobkova N.V. (1992). Dissociation of Multiple Behavioral Effects between Olfactory Bulbectomized C57Bl/6J and DBA/2J Mice. Physiol. Behav..

[39] Wu H.H., Wang S. (2010). Strain Differences in the Chronic Mild Stress Animal Model of Depression. Behav. Brain Res..

[40] O’Mahony C.M., Clarke G., Gibney S., Dinan T.G., Cryan J.F. (2011). Strain Differences in the Neurochemical Response to Chronic Restraint Stress in the Rat: Relevance to Depression. Pharmacol. Biochem. Behav..

[41] Gieryk A., Ziolkowska B., Solecki W., Kubik J., Przewlocki R. (2010). Forebrain PENK and PDYN Gene Expression Levels in Three Inbred Strains of Mice and Their Relationship to Genotype-Dependent Morphine Reward Sensitivity. Psychopharmacology.

[42] Brand T., Spanagel R., Schneider M. (2012). Decreased Reward Sensitivity in Rats from the Fischer344 Strain Compared to Wistar Rats Is Paralleled by Differences in Endocannabinoid Signaling. PLoS ONE.

[43] Deiana S., Fattore L., Spano M.S., Cossu G., Porcu E., Fadda P., Fratta W. (2007). Strain and Schedule-Dependent Differences in the Acquisition, Maintenance and Extinction of Intravenous Cannabinoid Self-Administration in Rats. Neuropharmacology.

[44] Marusich J.A., McCuddy W.T., Beckmann J.S., Gipson C.D., Bardo M.T. (2011). Strain Differences in Self-Administration of Methylphenidate and Sucrose Pellets in a Rat Model of ADHD. Behav. Pharmacol..

[45] Manduca A., Campolongo P., Palmery M., Vanderschuren L.J.M.J., Cuomo V., Trezza V. (2014). Social Play Behavior, Ultrasonic Vocalizations and Their Modulation by Morphine and Amphetamine in Wistar and Sprague-Dawley Rats. Psychopharmacology.

[46] Paré W.P. (2000). Investigatory Behavior of a Novel Conspecific by Wistar Kyoto, Wistar and Sprague-Dawley Rats. Brain Res. Bull..

[47] Ruda-Kucerova J., Zanda M.T., Amchova P., Fratta W., Fattore L. (2018). Sex and Feeding Status Differently Affect Natural Reward Seeking Behavior in Olfactory Bulbectomized Rats. Front. Behav. Neurosci..

[48] Stock H.S., Ford K., Wilson M.A. (2000). Gender and Gonadal Hormone Effects in the Olfactory Bulbectomy Animal Model of Depression. Pharmacol. Biochem. Behav..

[49] Dalla C., Pitychoutis P.M., Kokras N., Papadopoulou-Daifoti Z., Neill J.C., Kulkarni J. (2011). Sex Differences in Response to Stress and Expression of Depressive-Like Behaviours in the Rat. Biological Basis of Sex Differences in Psychopharmacology.

[50] Fattore L., Spano M.S., Altea S., Angius F., Fadda P., Fratta W. (2007). Cannabinoid Self-Administration in Rats: Sex Differences and the Influence of Ovarian Function. Br. J. Pharmacol..

[51] Fattore L., Spano M., Altea S., Fadda P., Fratta W. (2010). Drug- and Cue-Induced Reinstatement of Cannabinoid-Seeking Behaviour in Male and Female Rats: Influence of Ovarian Hormones. Br. J. Pharmacol..

[52] Castelli M.P., Fadda P., Casu A., Spano M.S., Casti A., Fratta W., Fattore L. (2014). Male and Female Rats Differ in Brain Cannabinoid CB1 Receptor Density and Function and in Behavioural Traits Predisposing to Drug Addiction: Effect of Ovarian Hormones. Curr. Pharm. Des..

[53] Pisanu A., Lo Russo G., Talani G., Bratzu J., Siddi C., Sanna F., Diana M., Porcu P., De Luca M.A., Fattore L. (2022). Effects of the Phenethylamine 2-Cl-4,5-MDMA and the Synthetic Cathinone 3,4-MDPHP in Adolescent Rats: Focus on Sex Differences. Biomedicines.

[54] Ruda-Kucerova J., Amchova P., Havlickova T., Jerabek P., Babinska Z., Kacer P., Syslova K., Sulcova A., Sustkova-Fiserova M. (2015). Reward Related Neurotransmitter Changes in a Model of Depression: An in Vivo Microdialysis Study. World J. Biol. Psychiatry.

[55] Kelly J.P., Wrynn A.S., Leonard B.E. (1997). The Olfactory Bulbectomized Rat as a Model of Depression: An Update. Pharmacol. Ther..

[56] Cabeza de Vaca S., Carr K.D. (1998). Food restriction enhances the central rewarding effect of abused drugs. J. Neurosci..

[57] Fattore L., Cossu G., Martellotta C.M., Fratta W. (2001). Intravenous self-administration of the cannabinoid CB1 receptor agonist WIN 55,212-2 in rats. Psychopharmacology.

[58] Ruda-Kucerova J., Amchova P., Babinska Z., Dusek L., Micale V., Sulcova A. (2015). Sex Differences in the Reinstatement of Methamphetamine Seeking after Forced Abstinence in Sprague-Dawley Rats. Front. Psychiatry.

[59] Anker J.J., Zlebnik N.E., Navin S.F., Carroll M.E. (2011). Responding during Signaled Availability and Nonavailability of Iv Cocaine and Food in Rats: Age and Sex Differences. Psychopharmacology.

[60] Contini A., Sanna F., Maccioni P., Colombo G., Argiolas A. (2018). Comparison between Male and Female Rats in a Model of Self-Administration of a Chocolate-Flavored Beverage: Behavioral and Neurochemical Studies. Behav. Brain Res..

[61] Jastrzębska J., Frankowska M., Suder A., Wydra K., Nowak E., Filip M., Przegaliński E. (2017). Effects of Escitalopram and Imipramine on Cocaine Reinforcement and Drug-Seeking Behaviors in a Rat Model of Depression. Brain Res..

[62] Gawlińska K., Jastrzębska J., Gamberini S., Gawliński D., Pieniążek R., Suder A., Wydra K., Frankowska M. (2020). The Impact of GABAB Receptors and Their Pharmacological Stimulation on Cocaine Reinforcement and Drug-Seeking Behaviors in a Rat Model of Depression. Eur. J. Pharmacol..

[63] Kelly J.P., Leonard B.E. (1996). Effects of Chronic Desipramine on Waiting Behaviour for a Food Reward in Olfactory Bulbectomized Rats. J. Psychopharmacol..

[64] Jastrzębska J., Frankowska M., Smaga I., Hubalewska-Mazgaj M., Suder A., Pieniążek R., Przegaliński E., Filip M. (2023). Evaluation of the 5-HT2C Receptor Drugs RO 60-0175, WAY 161503 and Mirtazepine in a Preclinical Model of Comorbidity of Depression and Cocaine Addiction. Pharmacol. Rep..

